# Pharmacologic immunomodulation via adenosine 2a receptor stimulation improves LV remodeling and systolic strain in regions adjacent to the infarct as assessed by cardiac MRI

**DOI:** 10.1186/1532-429X-18-S1-O73

**Published:** 2016-01-27

**Authors:** Ya-Jian Cheng, Elie R Chemaly, Yikui Tian, Frederick H Epstein, Brent A French

**Affiliations:** grid.27755.32000000009136933XBiomedical Engineering, University of Virginia, Charlottesville, VA USA

## Background

Agonists of the Adenosine 2a Receptor (A2aR) are used clinically for perfusion imaging, but they are also potent immunomodulators that reduce infarct size in animal models when administered at reperfusion. The long term effects of sustained A2aR agonist administration initiated after myocardial infarction (MI) are largely unexplored. We hypothesized that sustained administration of an A2aR agonist would inhibit post-infarct left ventricular (LV) remodeling and improve contractile strain in the LV.

## Methods

C57BL/6 mice were treated either with vehicle or a highly selective A2aR agonist (ATL313). All mice received 1h coronary occlusion and 28 days of reperfusion. ATL313 was administered for 28 days by subcutaneous micro-osmotic pumps implanted after MI. All mice underwent 7T CMR imaging at baseline and 2, 7, 14 & 28 days post-MI. CMR included short-axis black-blood cines covering the entire heart, with mid-ventricular cine DENSE (Displacement Encoding with Stimulated Echoes) for circumferential strain (Ecc). Late Gd-enhanced (LGE) inversion recovery imaging was performed on Days 2&7 and molecular imaging with a collagen-targeted Gd contrast agent (EP3533) was performed on Days 14&28. Mice with Day 2 LGE infarct sizes < 22% or >42% of LV mass were excluded from analysis.

## Results

Examples of LGE and DENSE strain mapping are shown in Panels A&B. Day 2 infarct size was similar between groups (ATL313 (n = 9): 35 ± 2 vs Vehicle (n = 8): 34 ± 2, mean ± SEM, p = NS). In Panel C, ATL313 significantly improved LV end-systolic volume as early as 2 days post-MI (mean ± SEM, *p < 0.05 vs Vehicle). In panel D, ATL313 improved Day 2 Ecc in adjacent zones vs. Vehicle (-10 ± 2 vs -6 ± 2%, *p < 0.05). In Panel E, ATL313 reduced LV mass at Day 28 vs Vehicle (121 ± 7 vs 143 ± 7 mg, *p < 0.05).

## Conclusions

Pharmacologic immunomodulation with an A2aR agonist inhibits LV remodeling, improves contractile strain in infarct-adjacent regions at Day 2 post-MI and reduces LV mass at Day 28. Combined with previous work, these results suggest that A2aR stimulation may prove beneficial in both acute and sub-acute MI.

**Figure 1:**
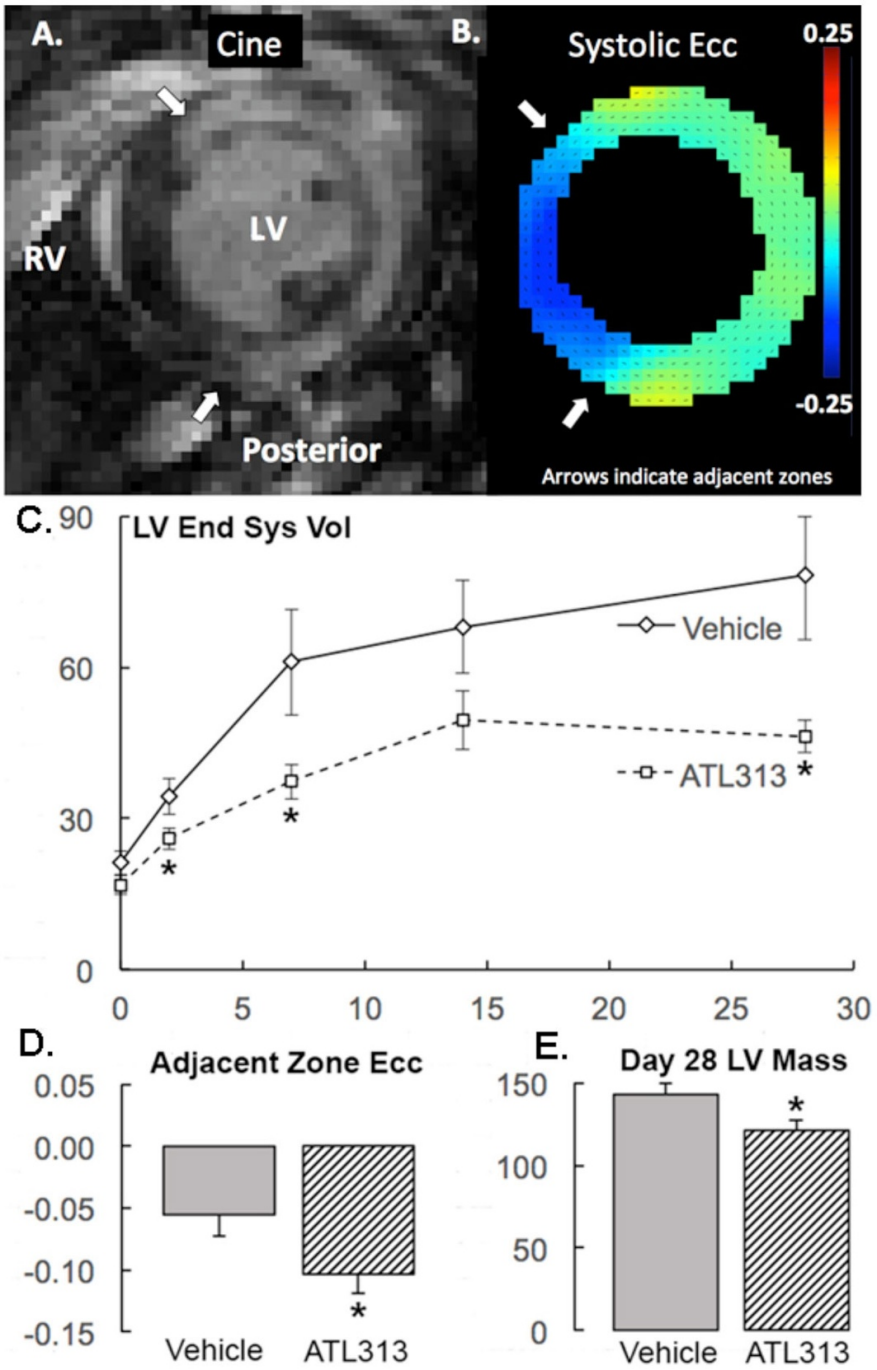
**A) Late Gd enhanced post-infarct mouse heart in short axis view**. LV: left ventricle, RV: right ventricle. B) Ecc mapping of the same mouse heart shown in Panel A. Arrows indicate adjacent zones next to the infarct. See Results for Panels C, D and E.

